# Preoperative Signs and Symptoms as Prognostic Markers in Nasal Septoplasty

**DOI:** 10.1155/2017/4718108

**Published:** 2017-08-13

**Authors:** Olga Shiryaeva, Magnus Tarangen, Caryl Gay, Liv Kari Døsen, Rolf Haye

**Affiliations:** ^1^Department of Quality, Lovisenberg Diakonale Hospital, Oslo, Norway; ^2^Department of Otorhinolaryngology, Lovisenberg Diakonale Hospital, Oslo, Norway; ^3^Faculty of Medicine, University of Oslo, Oslo, Norway

## Abstract

Identification of preoperative signs and symptoms that may predict the outcome of surgery is important, for both patient selection and the development of interventions for improving outcomes. The purpose of this study was to assess the value of some selected preoperative signs and symptoms for predicting outcomes of nasal septoplasty. Patients undergoing septoplasty with or without turbinoplasty responded to the Nasal Surgical Questionnaire (NSQ) preoperatively and six months postoperatively. The questionnaire contains visual analogue scales (VAS) for nasal obstruction during the day and at night. We compared preoperative and postoperative VAS scores in patients with unilateral versus bilateral septal deviation and patients with low versus high preoperative scores. Of 446 patients undergoing septoplasty from September 2014 to December 2015 who had responded to the preoperative NSQ, 286 (64.1%) also returned the postoperative version. There was greater improvement in obstruction in patients with preoperative unilateral compared to bilateral septal deviation (day scores, *p* = 0.04). The grade of deviation and the presence of concomitant bony conchal hypertrophy did not influence results. Patients with lower preoperative VAS scores obtained better end results than those with higher scores (*p* = 0.04). Type of septal deviation and preoperative VAS scores may aid in predicting outcome of nasal surgery.

## 1. Introduction

Patients, surgeons, and hospitals are interested in the results of surgery performed at their hospital. The results are not always predictable [1], but it would be helpful to surgeons to have data that might help in making a prognosis as to the outcome of surgery. This information may also be useful to patients in helping them decide whether to have nasal surgery as well as to set realistic expectations regarding likely outcomes. Studies have examined age [2–6], gender [3, 5–8], smoking habits [3, 5], allergy [3, 6, 9–13], prior surgery [6, 8], and preoperative obstruction scores [3, 5, 14] as predictive clues to the outcome of nasal surgery. Only a few studies have examined the type and grade of septal deviation [5, 15, 16]. As our primary objective, we aimed to assess the difference in the outcomes of patients with preoperative findings of unilateral versus bilateral septal deviation. Secondarily, we aimed to evaluate the outcomes in patients with lower versus higher preoperative nasal obstruction scores.

## 2. Material and Methods

The study was approved by the Ethics Committee of Lovisenberg Diakonale Hospital (LDH). Since 2014, LDH has performed quality control of nasal septoplasties with or without surgery to the inferior concha by using the Nasal Surgical Questionnaire (NSQ) to assess nasal symptoms [17]. The NSQ has both a preoperative version, which patients complete on the day of surgery, and a postoperative version, which is mailed to patients six months after surgery. The study population consists of patients aged 16 and older undergoing septoplasty with or without surgery to the inferior concha between September 2014 and December 2015. Patients undergoing any other nasal or sinus surgery as well as patients with any other nasal or sinus disease except nasal allergy were excluded. In the morning prior to surgery, the patients respond to the preoperative version of the NSQ. This contains separate visual analogue scales (VAS) assessing nasal obstruction during the day, at night, and during exercise. Each VAS has a 10 cm line, with the left end of the line (numbered 0) representing no obstruction and the right end of the line (numbered 10) representing complete obstruction. The patients are asked to rate their sense of nasal obstruction on each of the scales with a vertical line. The score is measured in mm from the left hand side of the scale. For other nasal symptoms and therapies, 4-point Likert scales are used with the following response categories: none, slight, moderate, and severe/daily use. In addition, questions about smoking habits and self-reported nasal allergy are included. Shortly after the operation the surgeon registers the following data into the surgical log: type of surgery, side (right, left, or bilateral) and grade of septal deviation (slight = less than half the width of the nasal cavity, moderate = half the width of the nasal cavity, and severe deviation = subtotal to total occlusion), turbinate hypertrophy (presence of conchal obstruction after the septum has been straightened), and septal crusting. The postoperative version of the NSQ (see questionnaire) contains an additional question about the surgery's degree of success (complete, substantial, slight improvement, unchanged, or worse) and is mailed to each patient five and a half months after surgery along with a prepaid return envelope. If it is not returned after three weeks, a reminder along with a second copy of the questionnaire is mailed to the nonrespondents. To compare the results after surgery for patients with high versus low preoperative scores, we chose to divide them into three groups of roughly equal size with an adequate spread in scores by using their preoperative VAS scores at night.

### 2.1. Statistical Analysis

Descriptive statistics (means and frequencies) were used to summarize sample characteristics and questionnaire responses. Independent sample *t*-tests were used to compare groups on continuous variables; Chi-square tests were used for group comparisons on categorical variables. Wilcoxon Signed Ranks Test was applied to estimate the differences between preoperative and postoperative VAS ratings for nasal obstruction both during the day and at night. Marginal homogeneity test was performed to estimate the differences between preoperative and postoperative ratings of nasal symptoms using a four-point scale. A significance level of *p* < 0.05 was used for all analyses. All analyses were conducted using SPSS for Windows, version 24.0 (IBM Corp, Armonk, NY).

## 3. Results

From September 2014 to December 2015, septoplasty with or without surgery to the inferior concha was performed on 446 patients who had completed the preoperative version of the NSQ. Of these, 286 (64.1%) responded to the mailed postoperative version of the NSQ and were included in this analysis. The sample had a mean age of 36.2 (±13.2) years and included 196 males (68.5%) and 90 (31.5%) females. There were 48 (16.8%) smokers and 88 (31.4%) reported having nasal allergy. Medication use for allergy symptoms was reported by 37 (12.9%) patients preoperatively and by 40 (14.0%) patients six months postoperatively. Severe sneezing or secretion was reported by 39 (13.6%) patients preoperatively and 30 (10.5%) postoperatively. Most patients were not allergic at the time they completed the questionnaires. Idiopathic rhinitis may therefore be responsible for the symptoms.

The preoperative, postoperative, and difference in VAS scores during the day and at night for the total sample and patient subgroups are shown in Table 1. There was no significant difference between pre- and postoperative Likert scores for crusting and use of medication. However, postoperative sneezing and secretion ratings improved significantly from preoperative status. There was no difference in the results between males and females nor between smokers and nonsmokers. The patients reporting nasal allergy had borderline less improvement in VAS scores (*p* = 0.06) than nonallergic patients. Older patients (≥36 years) obtained greater improvement in obstruction scores than younger ones (*p* = 0.01).

Our primary aim was to compare the results of patients with unilateral (*n* = 222) versus bilateral septal deviation (*n* = 64). The mean improvement in VAS scores was higher in patients with unilateral deviation compared to bilateral deviation (Table 2). This was significant for day scores. The degree of septal deviation did not influence the results (*p* = 0.81). Bony conchal hypertrophy was found in two-thirds of patients with unilateral septal deviation. There was no difference in preoperative, postoperative, or improvement in VAS scores between patients with or without conchal hypertrophy. Our secondary aim was to compare outcomes among patients with high versus low preoperative VAS scores. The differences between pre- and postoperative VAS scores according to preoperative VAS scores at night are shown in Table 3. Patients with high preoperative scores showed greater improvement in nasal obstruction, whereas patients with low preoperative scores reported the least obstruction postoperatively, which was significant at night (*p* < 0.05).

## 4. Discussion

In this prospective study, we found statistically and clinically significant improvement in nasal obstruction after septal surgery. Patients with unilateral septal deviation experienced better results than those with bilateral deviation. The improvement in VAS scores after surgery was higher in patients with high preoperative obstruction scores, but the end result (at least at night) was best for those with low preoperative scores.

The overall results from nasal septoplasty in this study are comparable to the studies reviewed by Rhee et al. [18]. We did not see any difference in results between males and females, which is consistent with most other studies [3, 5, 6, 8] except one [7] in which females had less improvement. Older patients showed better improvement than younger ones in our study, although other studies have found no differences by age [2–6]. Two studies [3, 5] reported that there was no difference in results between smokers and nonsmokers, as was our finding. There were an equal number of patients reporting presence of allergy at the time of surgery as at the postoperative assessment. However, due to medication many patients had only slight symptoms. Some studies report that there was no difference in results between patients with or without allergy [3, 6, 10, 11], while others found nonallergic patients show greater improvement [9, 12]. Our results indicate that nonallergic patients obtain slightly better improvement than allergic patients, although the results did not quite reach statistical significance (*p* = 0.06). Our study only differs from other ones in that older patients had better results than younger ones. We doubt, however, that this would significantly influence our primary and secondary results.

Our primary objective was to evaluate whether there was any difference in outcome for patients with unilateral versus bilateral septal deviation. Patients with unilateral deviation obtained better results than those with bilateral deviation. One study [15] examined six different types of septal deviation and found that septal surgery in types 2, 4, and 6 yielded the best results. This classification is difficult to undertake and we have tried to simplify it by categorising the deviation as uni- or bilateral. Their findings show that septal structural pathology has an impact on the results of surgery. This lends credit to our findings that patients with unilateral have a different prognosis than those with bilateral septal deviation.

Two studies [5, 16] have examined the impact the grades of septal deviation had on the outcome of septal surgery. They found, in accordance with our results, that the grade of deviation did not influence the results of surgery. Using CT scans pre- and postoperatively, a modest correlation was found between the degree of septal deviation and the improvement in VAS scores [13]. We believe that a complex structural deformity of the nasal septum is a greater challenge to surgeons than the degree of deviation.

Our secondary objective was to assess the prognostic importance of the preoperative VAS scores of obstruction. Two studies [5, 14] found that patients with a high degree of obstruction had greater improvement than those with less obstruction. They did not, however, mention the value of the end results. Another study [3] found the outcome to be best in patients with high preoperative obstruction scores. This may imply an increased improvement between pre- and postoperative scores or a better postoperative score. We found the improvement in obstruction scores to be greater in patients with high preoperative scores. However, the end results (the postoperative VAS scores) were lowest in patients with low preoperative scores, although only the night scores were significantly lower. Not surprisingly, there is a greater chance of obtaining greater improvements if the preoperative values are higher; however, to the patient it is likely the postoperative score that counts.

The strength of the study is that it is prospective and from one hospital with more than 200 patients. A potential drawback is the response rate of 64.1%.

## 5. Conclusion

We found that patients undergoing septoplasty with or without surgery to the inferior concha showed greater improvement, particularly during day, if the septal deviation was unilateral versus bilateral. Patients with high preoperative obstruction scores before surgery showed greater improvement after surgery than patients with low obstruction scores. However, patients with low preoperative obstruction scores had the lowest scores after surgery, particularly at night. These findings may be helpful to patients and their surgeons for setting appropriate expectations for surgical results. Additional studies are warranted to substantiate our findings.

## Supplementary Material

The postoperative version of the Nasal Surgical Questionnaire used for quality control of nasal septal surgery.

## Figures and Tables

**Table 1:** Preoperative, postoperative, and difference in mean VAS (SD) scores during day and at night for all patients and subgroups.

	Preoperative VAS score	Postoperative VAS score	Improvement in VAS score	*p* value^*∗*^
*Day*				
All patients	63.62 (19.54)	27.04 (23.13)	36.58 (27.45)	0.001
Allergy status				
Allergic	61.42 (17.49)	27.47 (20.54)	33.95 (24.58)	0.000
Nonallergic	63.36 (20.38)	24.05 (23.06)	39.31 (28.56)	0.000
Smoking status				
Smokers	67.94 (18.54)	29.23 (21.80)	38.70 (26.71)	0.000
Nonsmokers	62.75 (19.64)	26.60 (23.42)	36.15 (27.63)	0.000
Sex				
Males	63.24 (19.33)	27.57 (23.63)	35.66 (28.05)	0.000
Females	64.48 (20.08)	25.83 (22.09)	38.65 (26.08)	0.000
Age				
<36 years^*∗∗*^	63.43 (18.42)	30.47 (23.90)	32.95 (26.27)	0.000
≥36 years	63.86 (20.95)	22.63 (21.51)	41.23 (28.33)	0.000
*Night*				
All patients	75.84 (16.05)	33.75 (26.18)	42.09 (27.55)	0.002
Allergy status				
Allergic^#^	73.18 (16.58)	34.71 (22.69)	38.46 (25.83)	0.000
Nonallergic	76.07 (15.72)	30.37 (27.55)	45.69 (27.15)	0.000
Smoking status				
Smokers	78.15 (15.31)	35.17 (23.17)	42.97 (27.69)	0.000
Nonsmokers	75.39 (16.17)	33.46 (26.73)	41.92 (27.58)	0.000
Sex				
Males	74.43 (15.96)	33.24 (25.84)	41.18 (27.75)	0.000
Females	79.05 (15.62)	34.88 (26.94)	44.16 (27.16)	0.000
Age				
<36 years^*∗∗*^	74.73 (16.64)	36.64 (26.57)	38.08 (26.64)	0.000
≥36 years	77.28 (15.20)	30.02 (25.24)	47.26 (27.96)	0.000

^*∗*^Paired  test. Wilcoxon Signed Ranks Test (test comparing pre- and postoperative scores); ^*∗∗*^*p* < 0.01;  ^#^*p* = 0.06 (borderline significance), Mann-Whitney *U* test for difference in improvement between the two groups.

**Table 2:** Preoperative, postoperative, and difference in VAS scores (SD) for patients with unilateral (*n* = 222) versus bilateral (*n* = 64) septal deviation.

Deviation	Preoperative VAS score	Postoperative VAS score	Improvement in VAS score
*Day *			
Unilateral	63.91 (19.68)	25.88 (22.39)	38.39 (26.34)
Bilateral	61.84 (19.10)	31.70 (25.24)	30.33 (30.41)
*p* value	0.32	0.09	0.04^*∗*^
*Night*			
Unilateral	77.25 (15.58)	33.57 (26.46)	43.63 (26.99)
Bilateral	71.55 (16.90)	34.60 (25.25)	36.77 (29.04)
*p* value	0.007^*∗*^	0.61	0.09

^*∗*^ *p* value < 0.05, Mann-Whitney *U* test.

**Table 3:** Preoperative, postoperative, and improvement in mean VAS scores (SD) for groups divided according to preoperative VAS night scores.

	Preoperative VAS score ≤ 70	Preoperative VAS score 71–84	Preoperative VAS score 85–100	Total sample
Number of patients (%)	87 (30.5)	108 (37.9)	90 (31.6)	285 (100)
VAS day scores				
Preoperative^*∗*^	53.72 (12.91)	64.76 (17.22)	71.91 (23.29)	63.44 (19.53)
Postoperative	23.64 (20.97)	27.23 (23.94)	30.13 (23.85)	27.06 (23.09)
Pre-post difference^*∗*^	30.08 (23.05)	37.52 (27.90)	41.78 (29.79)	36.58 (27.45)
VAS night scores				
Preoperative^*∗*^	56.89 (12.88)	78.00 (3.75)	91.92 (4.96)	75.97 (16.03)
Postoperative^*∗*^	28.22 (22.90)	32.81 (25.87)	40.36 (28.37)	33.75 (26.18)
Pre-post difference^*∗*^	28.67 (24.10)	45.18 (26.33)	51.55 (27.42)	42.09 (27.55)

^*∗*^ *p* < 0.05, Mann-Whitney *U* test (significance between groups VAS ≤ 70 and VAS 85–100).
