# Supplementary material for: Preoperative Signs and Symptoms as Prognostic Markers in Nasal Septoplasty
Source: Int J Otolaryngol. 2017 Aug 13;2017:4718108. doi: 10.1155/2017/4718108 (PMC5572615; doi:10.1155/2017/4718108)
Supplement: Supplementary file 1 — The postoperative version of the Nasal Surgical Questionnaire used for quality control of nasal septal surgery. [file 4718108.f1.pdf]

Date _________________

Identification

After operation (answer when free of a cold/nasal infection)

NASAL SURGICAL QUESTIONNAIRE

Is your nasal breathing

☐ Completely improved ☐ Substantially improved ☐ Mildly improved ☐ Unchanged ☐ Worse

Rate your sense of obstruction

Put a mark on this scale

Open *(0=completely open. 10= completely blocked.)* **Blocked**

On a normal day	0--10
At night	0--10
During exercise	0--10

**Rate these nasal symptoms**	None	Slight	Moderate	Severe
Crusting	☐	☐	☐	☐
Bleeding	☐	☐	☐	☐
Sneezing	☐	☐	☐	☐
Secretion	☐	☐	☐	☐
Nasal pain	☐	☐	☐	☐

**Rate your use of nasal medication**	None	Slight	Moderate	Daily
Nonprescriptional nasal spray/drops (Naso/Nazaren/Otrivin/Rhinox/Zymelin/Zycomb)	☐	☐	☐	☐
Corticosteroid nasal spray/drops (Avamys/Budesonid/Flutide nasal/ Nasacort/Nasonex/Rhinocort)	☐	☐	☐	☐
Antihistamines (Aerius/Alzyr/Cetrizin/Clarityn/Kestine/ Loratadin/Telfast/Zyrtec/Xyzal)	☐	☐	☐	☐

Smoking ☐ None ☐1-10 daily ☐ 11 or more daily

Do you suffer from nasal allergy ☐ Yes ☐ No ☐ Uncertain

If yes
do you have nasal allergy at present ☐ Yes ☐ No

do you use allergy medication at present ☐ Yes ☐ No
